# Error in Figure

**DOI:** 10.1001/jamanetworkopen.2024.13426

**Published:** 2024-04-24

In the Original Investigation “P2Y12 Inhibitor Monotherapy vs Dual Antiplatelet Therapy After Deployment of a Drug-Eluting Stent: The SHARE Randomized Clinical Trial,”^1^ published March 7, 2024, there was an error in Figure 1. The number of patients enrolled and randomized should have been 1452. This article has been corrected.^1^
